# Avascular necrosis: radiological findings and main sites of involvement - pictorial essay

**DOI:** 10.1590/0100-3984.2017.0151

**Published:** 2019

**Authors:** Dair Jocely Enge Junior, Eduardo Kaiser Ururahy Nunes Fonseca, Adham do Amaral e Castro, Eduardo Baptista, Durval do Carmo Barros Santos, Laercio Alberto Rosemberg

**Affiliations:** 1 Department of Radiology and Diagnostic Imaging, Hospital Israelita Albert Einstein, São Paulo, SP, Brazil.; 2 Department of Diagnostic Imaging, Escola Paulista de Medicina da Universidade Federal de São Paulo (EPM-Unifesp), São Paulo, SP, Brazil.

**Keywords:** Osteonecrosis/diagnostic imaging, Radiography, Tomography, X-ray computed, Magnetic resonance imaging, Osteonecrose/diagnóstico por imagem, Radiografia, Tomografia computadorizada, Ressonância magnética

## Abstract

The term avascular necrosis describes any one of a number of bone diseases that have a common mechanism: the death of bone components due to lack of blood supply. Avascular necrosis can occur in diverse parts of the skeleton, each location-specific form not only receiving a distinct designation but also presenting unique epidemiologic characteristics. However, the imaging findings are similar in all of the forms, which pass through well-described radiological phases, regardless of the site of involvement. Because avascular necrosis can cause considerable morbidity if not properly detected and managed, the radiologist plays a fundamental role. The present study provides a brief review of the main radiological aspects of the various forms of avascular necrosis, illustrated on the basis of a collection of cases from our institution.

## Abstract (pt)

Necroses avasculares são um grupo de doenças ósseas que possuem como via comum a morte dos constituintes dos ossos por falta de suprimento vascular. Podem ocorrer nas mais diversas partes do esqueleto, recebendo não somente denominações distintas, como também apresentam epidemiologia única em cada uma dessas localizações. Seus achados nos métodos de imagem, no entanto, são similares, independente do seu sítio de acometimento, passando por fases radiológicas já bem descritas. Se não forem detectadas e manejadas de forma adequada, podem trazer grande morbidade aos pacientes, o que torna o papel do radiologista primordial. O presente estudo faz uma revisão sucinta acerca dos principais aspectos radiológicos das diversas formas de apresentação das necroses avasculares, ilustradas com base em uma coletânea de casos da nossa instituição.

## INTRODUCTION

Avascular necrosis, also known as osteonecrosis or aseptic necrosis, is a pathological process associated with a number of conditions and therapeutic interventions. In patients with direct damage to the bone vasculature (such as a femoral neck fracture) or direct lesion of bone components (such as radiation-induced damage), the cause can be clearly identified. However, in many patients, the mechanisms behind this disorder are not fully understood^(^^1^^-^^3^^)^.

Blood flow impairment leading to bone cell death seems to be common to most of the proposed etiologies of avascular necrosis. The process is usually progressive, resulting in ischemia and gradual bone destruction within a few months to two years in most patients^(^^1^^-^^3^^)^.

The exact prevalence of avascular necrosis is unknown. The ratio of male to female patients varies depending on the accompanying comorbidities^(^^2^^)^.

A number of traumatic and nontraumatic factors can contribute to the etiology of avascular necrosis. Preeminent among the traumatic factors are femoral neck fractures, whereas nontraumatic factors include the use of steroids, hemoglobinopathies, human immunodeficiency virus infection, alcoholism, smoking, and idiopathic, among other causes^(^^2^^)^.

## DISCUSSION

### Legg-Calvé-Perthes disease

**Figure 1:**
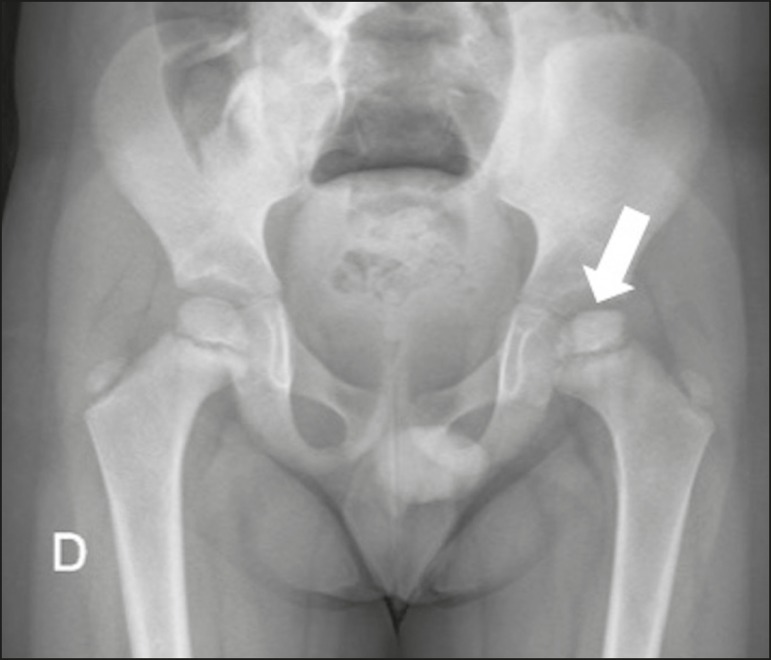
Asymmetry of the femoral heads, less pronounced on the left (arrow), with contour irregularity and areas of subchondral sclerosis.

**Figure 2:**
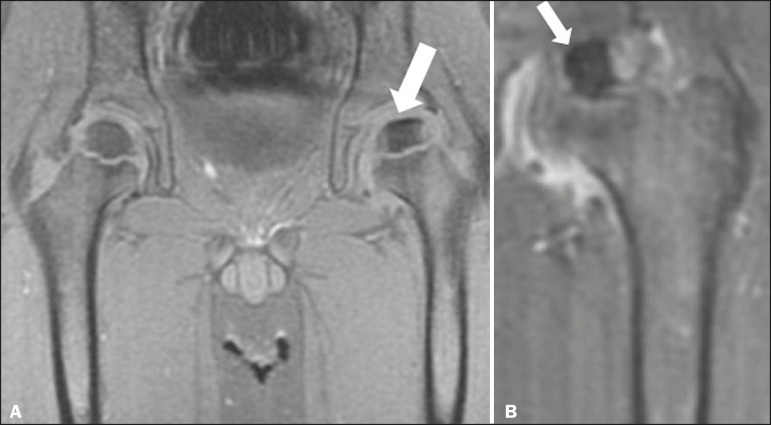
MRI of the same patient shown in Figure 1. **A:** Unenhanced T1-weighted sequence showing asymmetry of the femoral heads, less pronounced on the left, with contour irregularity and areas of subchondral sclerosis (arrow). There is contour irregularity and volume reduction of the ossification center of the left femoral head (epiphysis). **B:** Gadoliniumenhanced T1-weighted sequence showing an unenhanced sclerotic area (avascular necrosis) in the center (arrow).

In Legg-Calvé-Perthes disease, there is avascular necrosis of the femoral head epiphysis. It is most common in white males, its prevalence is highest among individuals between 5 and 7 years of age, and it is bilateral in 10-20% of patients^(^^4^^)^. Although its etiology is unknown, it is believed that the femoral head physis acts as a barrier to the blood supply of the epiphysis. Deformities and secondary osteoarthritis can develop^(^^4^^)^. The factors conferring a worse prognosis include the following^(^^4^^)^: older age at onset; lateral subluxation; involvement of more than 50% of the femoral head; neovascularization; fracture of the subchondral ossification center; metaphyseal and physeal plate signal abnormalities on magnetic resonance imaging (MRI); and neovascularization across the epiphysis, as illustrated in Figures 1 and 2.

### Kienböck's disease

**Figure 3:**
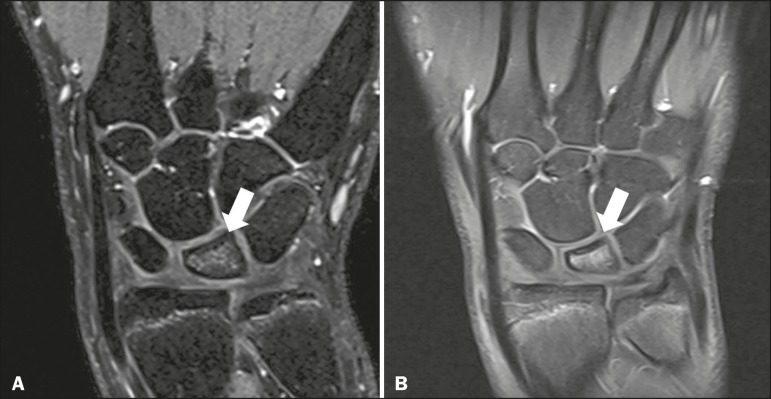
Contrast-enhanced T1- and T2-weighted MRI sequences, both fatsaturated, showing edema (**A**) and lunate bone marrow enhancement (**B**). The preserved morphology of the lunate bone suggests early-stage Kienböck's disease.

Kienböck's disease is characterized by avascular necrosis of the lunate bone (Figure 3). It is an insidious condition that affects the dominant wrist of young adults and is related to repetitive microtrauma^(^^5^^)^. The most common symptoms are pain in the dorsal surface of the wrist, mild edema, stiffness, and clicking^(^^5^^)^. Approximately 75% of cases have negative ulnar variance, which is defined as an ulna that is abnormally shorter than the radius^(^^5^^)^. Conservative treatment is highly effective in mild cases. As the disease progresses, there is sclerosis and fragmentation of the lunate. The most common surgical procedure used for the correction of negative ulnar variance is radial shortening. Proximal row carpectomy is a salvage procedure for refractory cases^(^^5^^)^.

### Kümmell disease

**Figure 4:**
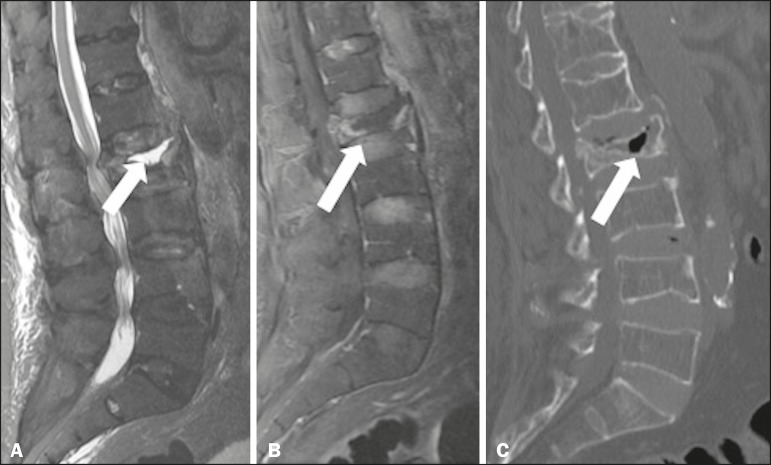
Gadolinium contrast-enhanced, fat-saturated T1- and T2-weighted MRI sequences (**A** and **B**, respectively) and computed tomography (**C**), showing fluid accumulation (**A**) and diffuse bone marrow enhancement of the L2 vertebral body (**B**). An unenhanced area is seen in the central and anterior regions of the vertebral body, surrounded by a fluid collection, suggesting avascular necrosis, collapse, height reduction, and displacement of the posterior wall of the L2 vertebral body, which contains gas foci (**C**) (arrow).

In Kümmell disease, there is post-traumatic avascular necrosis of the vertebral body secondary to ischemia caused by compressive fracture, with accumulation of intravertebral gas. It predominantly affects the lower thoracic or upper lumbar spine of elderly female patients with osteoporosis^(^^6^^)^. The condition can manifest as pain and kyphosis, progressing to vertebral collapse (Figure 4). Treatments include vertebroplasty and kyphoplasty^(^^6^^)^.

### Freiberg's disease

**Figure 5:**
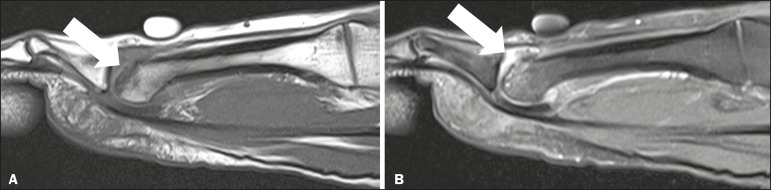
T1-weighted and fat-saturated T2-weighted MRI sequences showing chronic subchondral fracture/impaction in the dorsal aspect of the second metatarsal head (**A**), flattening of the joint surface, subchondral cysts, and bone edema (**B**), extending to the distal diaphysis. There is also metatarsophalangeal joint effusion with synovial thickening (arthritis).

In Freiberg's disease, there is avascular necrosis of the metatarsal head, most frequently of the second metatarsal bone (in 68% of cases). It is related to chronic repetitive trauma, systemic diseases (such as diabetes and systemic lupus erythematosus), and mechanical factors (such as the second metatarsal syndrome)^(^^7^^)^. It predominantly affects young women and manifests as pain and swelling of the metatarsophalangeal joints of the second toe^(^^7^^)^. The radiological findings vary depending on the stage of the disease. In the early stages, imaging exams may be normal. However, in more advanced stages, osteopenia can be seen in the center of the metatarsal head, with flattening of its contours, together with fragmentation and sclerosis. MRI findings include bone marrow edema, a serpentine line with low signal intensity near the metatarsal head, flattening of the contours of the metatarsal head, as well as sclerosis and fragmentation^(^^7^^)^, as can be seen in Figure 5.

### Köhler disease

**Figure 6:**
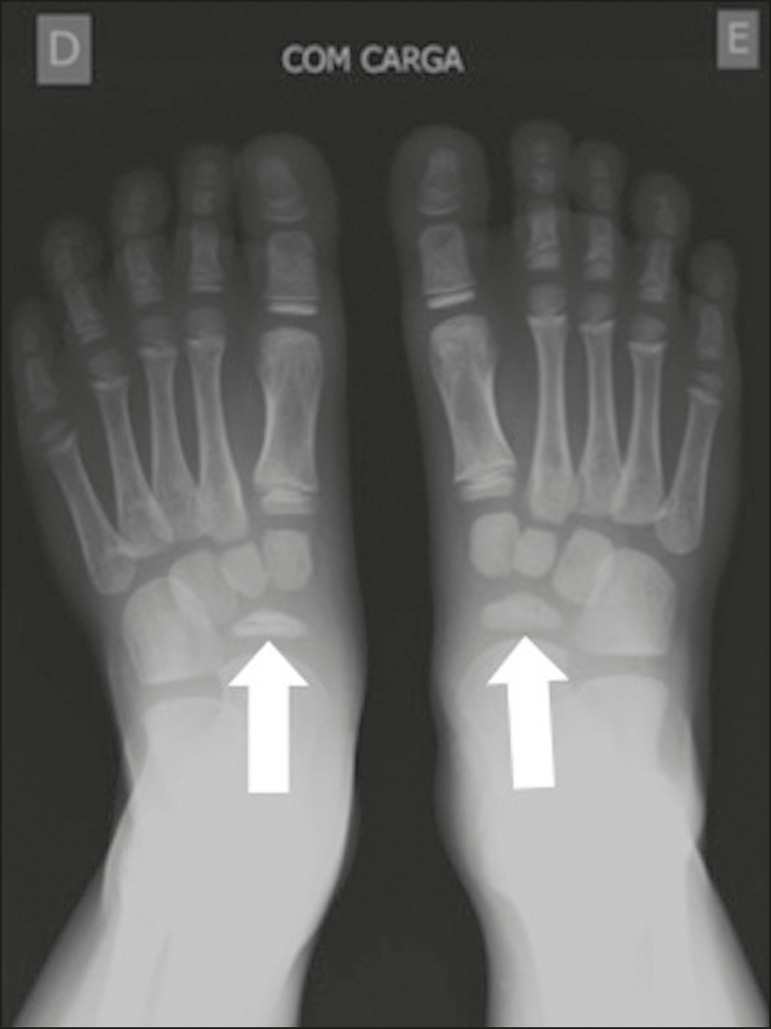
Sclerosis and volume reduction of the right navicular bone, consistent with avascular necrosis (arrow). On the contralateral side, the navicular bone is preserved (arrow).

**Figure 7:**
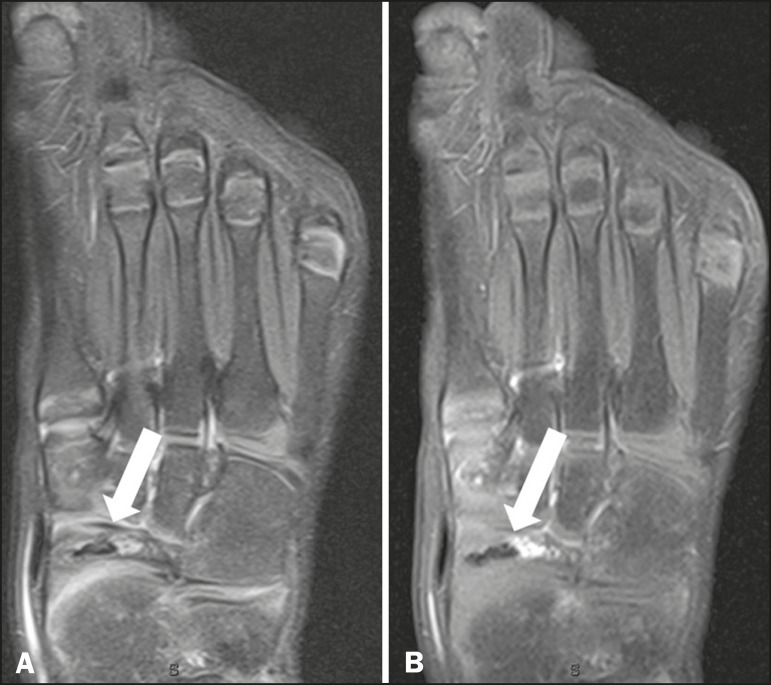
MRI of the same patient shown in Figure 6. **A:** T2-weighted image showing volume reduction and sclerosis of the navicular bone (arrow). **B:** Gadolinium-enhanced T1-weighted sequence showing foci of enhancement within and surrounding the bone, suggesting reactive hyperemia (arrow). Together, these findings suggest avascular necrosis.

In Köhler disease, there is avascular necrosis of the navicular bone. It is most prevalent in boys 4-6 years of age. It can be asymptomatic or can manifest as mild foot pain^(^^8^^)^. Imaging usually shows bilateral involvement starting at the lateral border of the navicular bone (Figures 6 and 7). In more advanced stages, there is fragmentation and sclerosis, as well as medial and dorsal subluxation of the medial aspect of the navicular bone^(^^8^^)^. It is a self-limiting condition, most patients achieving complete resolution of symptoms and restoration of their bone structure between 4 months and 4 years after the onset of the disease. If the pain persists for longer than expected, other causes (talocalcaneal coalition or accessory navicular bone) should be investigated^(^^8^^)^.

### Spontaneous osteonecrosis of the knee (SONK)

**Figure 8:**
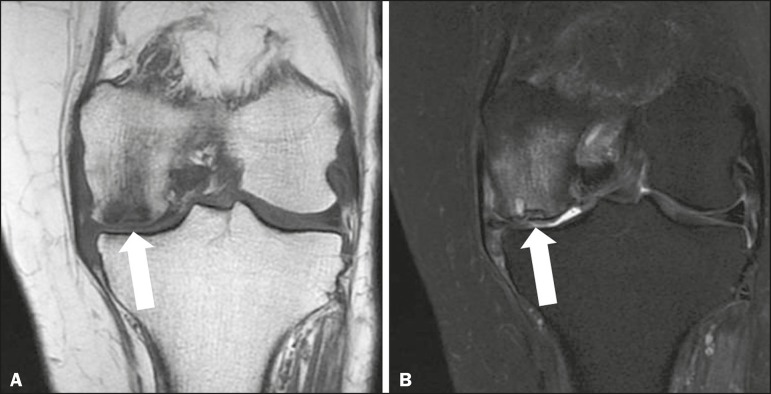
T1-weighted and fat-saturated T2-weighted sequences (**A** and **B**, respectively) showing subchondral fracture in the weight-bearing region of the medial femoral condyle, with impaction and flattening/deformity of the joint surface (**A**), accompanied by bone edema (**B**). There is a defined laminar geographic area consistent with secondary avascular necrosis and intense bone marrow edema, as well as subchondral cyst formation.

Spontaneous osteonecrosis of the knee (SONK), also known as Ahlback disease, there is spontaneous osteonecrosis of the knee. It most often affects white females in the sixth and seventh decades of life, presenting as sudden-onset knee pain that is not associated with local trauma or meniscal surgery^(^^9^^)^. It is almost always unilateral and usually affects the medial femoral condyle. It is often associated with a meniscal tear^(^^9^^)^. Radiological findings include an ill-defined, unenhanced area of severe edema in the femoral condyle, as well as a subchondral focus of low signal intensity related to a weight-bearing point (Figure 8). The prognosis and treatment depend on the size and extent of the subchondral lesion. If detected early and if the subchondral lesion is small (< 3.5 cm), clinical management is appropriate. If the lesion is large (> 50% of the femoral condyle or > 5.0 cm) or if clinical management results in no improvement, surgery is indicated^(^^9^^)^.

### Hass' disease

**Figure 9:**
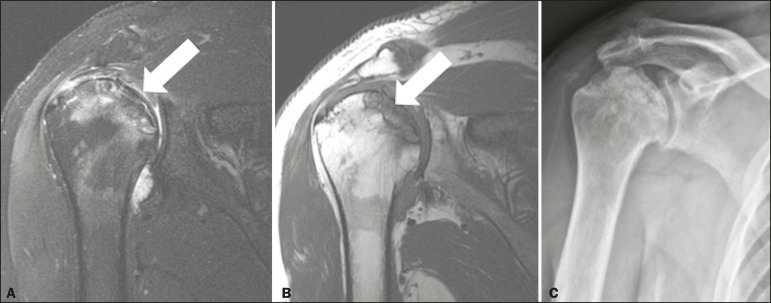
T2- and T1-weighted MRI sequences (**A** and **B**, respectively) showing the humeral head with a hypovascular area and a geographic pattern (avascular necrosis, arrow) affecting the superior region of the glenohumeral joint surface, and a subchondral fracture causing an osteochondral fragment in situ. There is also reactive bone marrow edema surrounding the necrotic area. An X-ray (**C**) showing heterogeneity of the humeral head with sclerotic areas in the superomedial region.

In Hass' disease, there is avascular necrosis of the humeral head, which is the second most common site of avascular necrosis. It affects the subchondral region and can lead to irregularities of the joint surface and to a consequent degeneration of the glenohumeral joint. Among the risk factors are the use of steroids and sickle cell disease^(^^10^^)^. The typical imaging findings of avascular necrosis are usually present (Figure 9). However, in the appropriate clinical context, the classic crescent sign is diagnostic of the condition^(^^10^^)^.

### Dias disease

**Figure 10:**
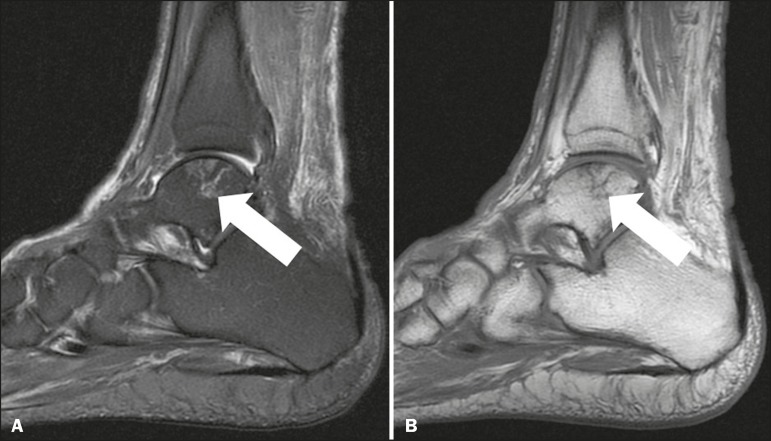
T1-weighted and fatsaturated T2-weighted MRI sequences showing an area with a geographic pattern and signal changes in the central portion of the lateral talar dome (arrow), consistent with bone infarction. The contours and morphology of the articular surface of the talar dome are preserved.

In Dias disease, there is avascular necrosis of the talus, which can be related to traumatic or nontraumatic events (such as the use of steroids and sickle cell disease). The post-traumatic etiology is seen in cases of fractures, especially of the talar neck. In those cases, the Hawkins classification is used to estimate the risk of fracture progression to avascular necrosis. The blood supply of the talus runs from its neck to its body and is most abundant in the medial aspect. Radiologically, it can manifest as irregularities of the talar dome (Figure 10), although the finding of serpiginous borders with a fatty core is a hallmark^(^^11^^)^.

## CONCLUSION

Avascular necrosis can occur in various parts of the skeleton. However, the imaging findings are similar in all of the forms, which pass through well-described radiological phases, regardless of the site of involvement. If not properly detected and managed, it can cause considerable morbidity, often progressing to secondary osteoarthrosis, which can require surgical treatment.
